# Cholesteryl hemiazelate identified in CVD patients causes in vitro and in vivo inflammation

**DOI:** 10.1016/j.jlr.2023.100419

**Published:** 2023-07-21

**Authors:** Neuza Domingues, Joana Gaifem, Rune Matthiesen, Diana P. Saraiva, Luís Bento, André R.A. Marques, Maria I.L. Soares, Julio Sampaio, Christian Klose, Michal A. Surma, Manuel S. Almeida, Gustavo Rodrigues, Pedro Araújo Gonçalves, Jorge Ferreira, Ryan Gouveia e Melo, Luís Mendes Pedro, Kai Simons, Teresa M.V.D. Pinho e Melo, M. Guadalupe Cabral, Antonio Jacinto, Ricardo Silvestre, Winchil Vaz, Otília V. Vieira

**Affiliations:** 1 iNOVA4Health, NOVA Medical School, Faculdade de Ciências Médicas, (NMS, FCM), Universidade Nova de Lisboa, Lisboa, Portugal; 2 Life and Health Sciences Research Institute (ICVS), School of Medicine, University of Minho, Portugal and ICVS/3B's, PT Government Associate Laboratory, Braga/Guimarães, Portugal; 3 Department of Chemistry, Coimbra Chemistry Centre, Institute of Molecular Sciences, University of Coimbra, Coimbra, Portugal; 4 Lipotype GmbH, Dresden, Germany; 5 Hospital Santa Cruz, Centro Hospitalar de Lisboa Ocidental, Carnaxide, Portugal; 6 Department of Vascular Surgery, Hospital de Santa Maria, Centro Hospitalar Universitario Lisboa Norte (CHULN), Lisboa, Portugal

**Keywords:** cholesteryl hemiesters, cholesteryl hemiazelates, lipidomics, innate inflammatory responses, atherosclerosis

## Abstract

Oxidation of PUFAs in LDLs trapped in the arterial intima plays a critical role in atherosclerosis. Though there have been many studies on the atherogenicity of oxidized derivatives of PUFA-esters of cholesterol, the effects of cholesteryl hemiesters (ChEs), the oxidation end products of these esters, have not been studied. Through lipidomics analyses, we identified and quantified two ChE types in the plasma of CVD patients and identified four ChE types in human endarterectomy specimens. Cholesteryl hemiazelate (ChA), the ChE of azelaic acid (*n*-nonane-1,9-dioic acid), was the most prevalent ChE identified in both cases. Importantly, human monocytes, monocyte-derived macrophages, and neutrophils exhibit inflammatory features when exposed to subtoxic concentrations of ChA in vitro. ChA increases the secretion of proinflammatory cytokines such as interleukin-1β and interleukin-6 and modulates the surface-marker profile of monocytes and monocyte-derived macrophage. *In vivo*, when zebrafish larvae were fed with a ChA-enriched diet, they exhibited neutrophil and macrophage accumulation in the vasculature in a caspase 1- and cathepsin B-dependent manner. ChA also triggered lipid accumulation at the bifurcation sites of the vasculature of the zebrafish larvae and negatively impacted their life expectancy. We conclude that ChA behaves as an endogenous damage-associated molecular pattern with inflammatory and proatherogenic properties.

The underlying cause of most CVDs is atherosclerosis, a chronic progressive inflammatory pathology characterized by subintimal lipid accumulation in the vascular wall that can progress for years without symptoms (1, 2). The relationship between LDL cholesterol and risk of CVD has been well established through numerous epidemiological, observational and genetic studies, and interventional clinical trials (2, 3). Since decades, oxidation of accumulated unsaturated lipids in the arterial intima has been believed to be the cause of most of the pathological consequences of atherosclerosis (4, 5, 6, 7, 8, 9, 10). Though generalized targeting of lipid peroxidation with antioxidants has not proven successful in the prevention of CVD in humans (11, 12), the fact that transgenic expression of an antibody to oxidized phospholipids (oxPLs) suppresses lesions in hypercholesterolemic mice argues in favor of lipid oxidation playing a critical role in atherosclerosis (13). Indeed, a large body of evidence suggests that the oxidation of PUFAs in LDL trapped in the vessel wall produces proinflammatory lipid species leading to *1*) expression of adhesion molecules and chemotactic factors that contribute to the recruitment of circulating monocytes into the intimal space; *2*) inhibition of the ability of resident macrophages to leave the intima; *3*) enhanced lipoprotein uptake rate, which leads to lysosomal lipid accumulation and to “pathogenic” foam cell formation; and *4*) increased cell death and consequent loss of endothelial integrity, leukocyte recruitment, and inflammation (6, 14, 15).

Among the oxidized LDL (oxLDL) components, oxPLs, oxidized PUFA-esters of cholesterol and oxysterols have been the major focus of several studies in the field, resulting in the identification of molecular structures, receptors, and signaling pathways associated with inflammatory responses in vascular cells (9, 16, 17, 18, 19, 20, 21, 22, 23). However, in all these studies, very little or no attention has been given to cholesteryl hemiesters (ChEs), the end products of oxidation of the PUFA-esters of cholesterol. To the best of our knowledge, we (24) were the first to address the possible role of ChEs in atherogenesis by using cholesteryl hemisuccinate (ChS), a ChE that does not appear either in the plasma or atheromata of patients with atherosclerosis.

LDLs transport cholesteryl esters (CEs), about 67% of which are PUFA esters with, on average, 57% of cholesteryl linoleate and 10% of cholesteryl arachidonate (24). These PUFA esters are oxidized in human atherosclerotic lesions (25, 26). Their oxidative scission leads to the formation of oxoesters of cholesterol, which have been identified in the “core aldehyde” fraction of ex vivo samples of human atheromata (25) and also in oxLDL (27), and finally to the corresponding ChE. ChEs are amphiphilic and have a negative charge at physiological pH. Similarly to what has been demonstrated for cholesterol (28), ChE may also be expected to equilibrate through passive diffusion between the oxLDL (where they are formed) and the aqueous (extracellular and intracellular) phases, as well as all membranes of neighboring cells. However, despite the well-established presence of their precursors in the atheroma, the role of ChE in atherogenesis is still unclear. In our previous work, we showed that a commercially available, but physiologically irrelevant ChE, ChS, is sufficient to cause irreversible lysosomal lipid accumulation (lipidosis) and is toxic to macrophages (24). In primary mouse bone marrow-derived macrophages, ChS exposure increased the secretion of interleukin-1β (IL-1β), TNF-α, and IL-6 (29). All these features are typically seen in atherosclerosis. In addition, zebrafish larvae fed with a ChS-enriched diet exhibited lipid accumulation, myeloid cell infiltration into the vasculature, and decrease in life expectancy (29). Interestingly, under the same conditions, the effects of ChS were more profound than the effects of free cholesterol (FC) (29). More recently, we synthesized cholesteryl hemiazelate (ChA), a cholesteryl-9-oxononanoate oxidation product, that is expected to be formed when cholesteryl linoleate undergoes oxidation (24). ChA-treated vascular smooth muscle cells acquire a foam-cell-like phenotype and exhibit membrane fluidity alterations (30). Furthermore, in ChA-treated macrophages, the dysfunctional lysosomes full of undigested cargo are exocytic. This outcome can be implicated in the initiation and perpetuation of inflammation in atherosclerosis (31).

In the present work, we show that ChEs are found in the plasma of CVD patients at average concentrations considerably higher than in age-matched controls as well as in carotid atheroma plaques. We also demonstrate the role of the most prevalent ChE found in these human tissues, ChA, as a potent inducer of innate inflammatory responses. Importantly, our findings show that ChA changes the inflammatory profile of human monocytes and macrophages. In zebrafish larvae, ChA induces the early features of atheroma formation, such as lipid accumulation and myeloid cell infiltration into their vasculature. Finally, pharmacological approaches indicate that in zebrafish larvae inflammasome as well as the lysosome protease cathepsin B are involved in the inflammatory process. Together, our data indicate that ChA behaves as an endogenous damage-associated molecular pattern with inflammatory and proatherogenic properties.

## Materials and Methods

### Plasma and carotid endarterectomy specimens

Blood samples were obtained from CVD patients and healthy individuals after explaining the purpose of the study and obtaining written informed consent from them or their legal representatives. The entire process was approved by the Ethical Review Board of the Faculty of Medicine of the NOVA University of Lisbon and the Ethics Committee for Health of the Centro Hospitalar de Lisboa Ocidental, Hospital Santa Cruz. All experiments were performed in accordance with the guidelines and regulations. The details for the different cohorts used in this study are described in supplemental Table S1. Blood samples were drawn from nonfasting donors. The blood was collected into tubes containing an anticoagulant (heparin or EDTA). More details on the sample preparation have been described elsewhere (32, 33). After collection, plasma samples were immediately frozen at −80°C.

Carotid atheroma plaques were isolated from patients with carotid artery disease submitted to open endarterectomy at Hospital Santa Maria, Centro Hospitalar Universitário Lisboa Norte. Inclusion criteria were symptomatic carotid artery stenosis of 50–99% or asymptomatic carotid artery disease of 70–99% (measured with ultrasound). These samples constitute discarded tissue generated during medical procedures and were collected from patients after informed consent. The excised carotid atherosclerotic plaques were rapidly dissected into maximally diseased atherosclerotic regions and into regions devoid of disease and placed in liquid nitrogen for shotgun lipidomics. Only the necrotic cores of those tissues were processed for shotgun lipidomics. The entire process was approved by the Joint Bioethics Committee of the Faculty of Medicine (University of Lisbon) and Centro Hospitalar Universitário Lisboa Norte (ref^a^ 209/18, de 27th of July 2018).

All experiments were performed in accordance with the guidelines and regulations including, the Universal Declaration on Bioethics and Human Rights of UNESCO, 2005; The Charter of Fundamental rights of the EU, 2012; Ethical principles for medical research involving human subjects—Declaration of Helsinki, 2013; and EU Regulation 2016/679 and Good Clinical Practice guidelines (Directive 2001/20/EC) and EU Clinical Trials Directive (2005/28/EC). Moreover, they complied with national legislation for the scientific use of human biological samples (Law Nº 12/2005 and Nº 131/2014).

### Synthesis of ChEs

ChEs were prepared following a general procedure described in the literature for the synthesis of ChS (34). ChA was synthesized from the reaction of commercially available cholesterol with freshly prepared azelaic anhydride, which was obtained by reacting azelaic acid with acetyl chloride, as described elsewhere (30, 35). Cholesteryl hemiglutarate (ChG), the ChE of glutaric acid (*n*-pentane-1,5-dioic acid), was prepared by the reaction of commercially available cholesterol with 1.7 M equivalents of commercially available glutaric anhydride in dry pyridine under reflux for 6 h (supplemental Fig. S1). Trituration of the crude product with methanol, followed by purification by flash chromatography with chloroform/methanol/ammonia (50:5:0.25), gave the target ChG as a white solid in 37% yield. The degree of purity for ChA and ChG is >99%. The detailed experimental procedure and characterization data for ChA and ChG are available in the supplemental data section.

### Lipid extraction, MS lipidomics, data acquisition, and analysis

MS-based lipid analysis was performed at Lipotype GmbH (Dresden, Germany) as previously described (36). Briefly, 50 μl of diluted plasma (equivalent to 1 μl of undiluted plasma) was mixed with 130 μl of 150 mM ammonium bicarbonate solution, and 810 μl of methyl tert-butyl ether/methanol (7:2, v/v) was added. About 21 μl of an internal standard mixture was premixed with the mixture of organic solvents. The internal standard mixture covered the major lipid classes present in plasma as described previously (32). In addition, 100 pmol per sample of ChS was added for quantification of ChE. The plate was then sealed with a teflon-coated lid, shaken at 4°C for 15 min, and spun down (3,000 *g*, 5 min) to facilitate separation of the liquid phases. About 100 μl of the organic phase was transferred to an infusion plate and dried in a speed vacuum concentrator. Dried lipids were resuspended in 40 μl of 7.5 mM ammonium acetate in chloroform/methanol/propanol (1:2:4, v/v/v), and the wells were sealed with an aluminum foil to avoid evaporation and contamination during infusion. All liquid handling steps were performed using a Hamilton STARlet robotic platform with the Anti Droplet-Control feature for pipetting of organic solvents. Samples were analyzed by direct infusion in a QExactive mass spectrometer (Thermo Fisher Scientific) equipped with a TriVersa NanoMate ion source (Advion Biosciences). About 5 μl were infused with gas pressure, and voltage was set to 1.25 psi and 0.95 kV, respectively. We scanned for the *m/z* 400–650 in Fourier transform MS − (Rm/z = 200 = 280,000) for 15 s with lock mass activated at a common background (*m/z* = 529.46262) to detect ChE as deprotonated anions. Automatic gain control was set to 106, and ion trap filling time was set to 50 ms. ChEs were identified and quantified by their accurate intact mass from the Fourier transform MS spectra with Lipotype Xplorer, a proprietary software developed from LipidXplorer (37, 38). In the CEA samples, for the identification of the various ChE species, 50 mg of atheromal “gruel” was homogenized in 1 ml of MS water, and the lipid content of the suspension was extracted as described for the blood plasma.

### Preparation of liposomes

POPC and ChA were mixed at a 35:65 M ratio. The detailed protocol for liposome preparation was described previously by our group (24). POPC liposomes were always used as control.

### Blood-derived monocyte isolation and in vitro differentiation into macrophages

Experiments were performed as previously described (39), using buffy coats isolated from healthy donors (six to eight different donors) supplied by the Hospital of Braga, after approval of the Competent Ethics Committee. The human samples received were handled in accordance with the guidelines approved by the Competent Ethics Committee. All the donors agreed and signed an authorized consent (ethical approval reference SECVF014/2015). After buffy-coat preparation, monocytes were isolated by centrifugation using Histopaque®-1077 (Sigma-Aldrich) followed by immunomagnetic separation using a human anti-CD14 purification kit (Miltenyi Biotec). The cell purity was always confirmed by flow cytometry and was superior to 95%. Purified monocytes were then directly used or differentiated in vitro into monocyte-derived macrophage (MDM). Cells were cultured in RPMI-1640 medium containing heat-inactivated FBS (10% FBS; Gibco), l-glutamine (2 mM; Gibco), penicillin (50 U ml^−1^; Gibco), streptomycin (50 μg ml^−1^; Gibco), and Hepes (10 mM; Gibco), and supplemented with the human macrophage colony-stimulating factor (20 ng/ml M-CSF; PeproTech) for 7 days at 37°C under a humidified 5% CO_2_ air atmosphere. On the third day, new medium was added to the cell culture in order to supply them with new nutrients. In experiments where the ChA effect on monocyte differentiation into macrophages was tested, the incubation media with M-CSF were supplemented with subtoxic concentrations of ChA (10 or 25 μM). After differentiation, cell culture was removed for cytokine quantification by ELISA using commercially available kits (BioLegend) and according to the manufacturer’s instructions.

### Isolation and culture of neutrophils

Neutrophils were isolated from the peripheral blood collected in EDTA tubes of five healthy donors. To separate the neutrophils, whole blood was layered on top of a solution of Histopaque H1117 and H1009 (Sigma-Aldrich) and centrifuged for 20 min at 2000 rpm with brake off. After centrifugation, the neutrophil layer was collected into a new tube. Lysis of red blood cells was performed with red blood cell lysis buffer (BioLegend) for 15 min at room temperature. Isolated neutrophils were then washed once with HBSS (Gibco) and cultured on a 96-well plate with U bottom (Sigma-Aldrich) in RPMI-1640 (Gibco) supplemented with 1% autologous plasma. Neutrophils were maintained 4 h in culture with Brefeldin A (BioLegend) to stop exocytosis.

### Flow cytometry

MDMs were detached by incubation with TrypLE™ Express solution (Life Technologies) at 37°C for 10 min. For the analysis of surface markers, monocytes and MDMs were incubated for 20 min with saturating concentrations of monoclonal antibodies against HLA-DR (BioLegend; clone L243), CD86 (BioLegend; clone IT2.2), CD206 (BioLegend; clone 15-2), and CD163 (BioLegend; clone GHI/61). Cells were also stained with dihydrorhodamine 123 and 4-amino-5-methylamino-2',7′-difluorofluorescein diacetate, all from Invitrogen.

For experiments with neutrophils, cells were stained with BD Horizon^TM^ Fixable Viability Stain 450 (BD Biosciences), followed by the surface monoclonal mouse antihuman-conjugated antibodies: anti-CD15-PE (clone H198) and anti-CD11b-FITC (ICRF44) from BioLegend. Intracellular staining was performed after fixation and permeabilization with Fix/Perm kit (eBiosciences), with the antibodies anti-IL-1β-FITC (clone JK1B-1), anti-IL-6-APC (MQ2-13A5), anti-TNFα-APC (Mab11) from BioLegend, and anti-IL-10-FITC (BT-10) from eBioscience.

### Zebrafish maintenance and feeding

The animal study was reviewed and approved by Animal User and Ethical Committees at NMS Research—NOVA Medical School and the Portuguese National Authority for Animal Health (DGAV). Zebrafish lines wild-type zebra fish (AB), *Tg*(*fli1:EGFP*), *Tg*(*pu.1:EGFP*), and *Tg*(*mpeg.mCherryCAAX SH378*, *mpx:EGFP i114*), the latter was obtained from the Sheffield University, were maintained in a recirculating system with a 14 h/day and 10 h/day-night cycle at 28°C. Zebrafish embryos were obtained by in vitro fertilization and kept in E3 zebrafish embryo medium at 28°C until reaching the desired developmental stage. Zebrafish larvae were fed twice a day for 10 days, starting at the fifth day postfertilization (dpf), with control diet (GEMMA Micro 75 from Skretting as the standard zebrafish larval food; pellet size = 50–100 μm; analytical constituents: crude protein 59%, fat 14%, ash 14%, fiber 0.2%, and phosphorus 1.3%), FC-enriched diet (normal food supplemented with FC), and ChA-enriched diet (normal food supplemented with 3% ChA w/w or 52 μmol per gram of food). The food was prepared as previously described (29, 40). For evaluation of vascular lipid accumulation, the food was also supplemented with 10 μg/g red fluorescent CholEsteryl BODIPY® 542/563 C11 (Molecular Probes).

For the experiments with caspase-1 and cathepsin B inhibitors, 6 dpf SH378 zebrafish larvae were fed overnight with normal diet, FC-enriched (normal food supplemented with 10% FC, w/w, corresponding to 260 μmol per gram of food), and ChA-enriched diet (normal food supplemented with 14% ChA w/w, corresponding to 260 μmol per gram of food). Caspase-1 inhibitor 50 μM (c-YVAD-AOM; Calbiochem) and 50 μM cathepsin B inhibitor (ZRLR, Poland kindly given by Ewa Wieczerzak, Department of Biomedicinal Chemistry, Faculty of Chemistry, University of Gdansk) were added into the E3 embryonic media 1 h before feeding and were present throughout the experiment.

### Microinjection of lipids

ChA, FC, and POPC liposomes were diluted to the desired concentrations in sterile PBS to be injected into the circulation of zebrafish larvae. POPC liposomes were used as control. The microinjection plate was a Petri dish filled with two layers of 2% agarose, one flat to act as a base for the second layer that has gutters (small trenches) on the surface to hold the larvae in place. The needles were provided by the fish facility staff. In these, we inserted 2.5 μl of the lipid emulsion. To calibrate the volume of liquid that would be injected, we used a slide marked with a microscale and a drop of oil. The tip of the needle was broken with forceps until the drop of the liquid in the oil had the desired diameter. Larvae were then anesthetized with MS-222 (Tricaine) and placed in the Petri dish with the gutters. The larvae were aligned in a way that the head was facing away from the user and the dorsal side was facing the needle. For the injection, the needle at an angle of about 30° was inserted slowly in the posterior cardinal vein, which leads directly to the heart, and the solution (1.5–2 nl) was injected. The injection was considered successful when a noticeable increase in flow of fluid was observed in the heart.

### Cell sorting

Larvae were sacrificed on ice to maintain cell viability and transferred to PBS containing 0.1 mg/ml liberase. Tissues were digested at 28°C for 20–30 min and mechanically dissociated by resuspending the tissue every 5 min. The cell suspension was passed through a CellTrics 30 μm filter directly to a new Eppendorf, and heat-inactivated FBS was added (10% final concentration). The cell suspension was then sorted in a FACSAriaIII^TM^ high speed cell sorter (Becton Dickinson) equipped with a 561 nm laser (50 mW solid state) and a 630/75 nm BP for mCherry excitation. PBS was used as sheath fluid and run at a constant pressure of 207 kPa with a 100 μm nozzle and a frequency of drop formation of approximately 30 kHz. Cells were directly collected into individual wells of a Nunc™ Lab-Tek™ chambered plate and allowed to adhere at room temperature for 2 h.

### Staining of lysosomes and neutral lipids

After cell sorting, macrophages were incubated for 20 min with BODIPY 493/503 (1:500 dilution) and LysoTracker (1:1,000 dilution) and imaged under a confocal microscope. Alternatively, zebrafish larvae, at 15 dpf, were incubated in a 1:100 dilution of LysoTracker Green in E3 medium at 28.5°C for 45 min to 1 h, washed twice, and then imaged (live imaging).

### Confocal imaging and analysis

At the time points indicated in the figure legends, zebrafish larvae were fixed in 4% paraformaldehyde and mounted on a coverslip using mounting media (1 g of DABCO in 10 ml PBS and 40 ml glycerol) prior to imaging. For live imaging, animals were anesthetized with tricaine methane sulfonate and mounted in 1% low melting point agarose in E3 medium. A Zeiss LSM710 confocal microscope was used, and optical sections in the tail area were acquired with a 40× objective.

For quantification of myeloid cells in the caudal vein, fluorescent cells were counted and normalized to the length of the analyzed segment of the vasculature. To quantify lipid accumulation in the vasculature, ImageJ software (National Institutes of Health and the Laboratory for Optical and Computational Instrumentation [LOCI, University of Wisconsin]) was used. The lipid structures were delineated, and the area was measured. The lipid intensity was normalized to the total area of the vasculature analyzed.

For cell live imaging, cells were imaged in a Nunc™ Lab-Tek™ chambered plate using the water 40× objective. The area and intensity of lysosomes and neutral lipids were measured using ImageJ software.

### Quantitative RT-PCR

Total RNA was extracted with Trizol reagent according to the manufacturer's instructions. RT was performed using the NZY first-strand cDNA synthesis kit (NZYtech). Quantitative PCR was performed in a 96-well optical plate using SYBR green master mix (NZYtech). PCR and data acquisition were performed using the AB7300 Real-Time PCR thermal cycler with *Step One software* (v2.2.2; Applied Biosystems). *eef1al1* (eukaryotic elongation factor 1 alpha like 1) and *rpl13a* (ribosomal protein L13a) were used as housekeeping genes to normalize the mRNA expression levels. Target gene expression was determined by relative quantification (ΔΔCt method) to the housekeeping reference genes and the control sample.

The following zebrafish forward and reverse primers were used:

*v-cam1* (TTGCAGTTGTTTCCCACACG; CCTAACGCGGTCCAGACAAA),

*il-1β* (GTAACCTGTACCTGGCCTGC; AACAGCAGCTGGTCGTATCC),

*il-6* (ACGTGAAGACACTCAGAGACG; CGTTAGACATCTTTCCGTGCTG),

*tnf-α* (CAGGGCAATCAACAAGATGG; TGGTCCTGGTCATCTCTCCA),

*il-10* (GCTCTGCTCACGCTTCCTTC; TGGTTCCAAGTCATCGTTGT)

*eef1a1l1* (CCTTCAAGTACGCCTGGGTGTT; CACAGCACAGTCAGCCTGAGAA),

*rpl13a* (TGACAAGAGAAAGCGCATGGTT; GCCTGGTACTTCCAGCCAACTT).

### Zebrafish survival evaluation

Zebrafish survival was first assessed in 5 dpf larvae fed with different diets for 10 days. Larvae were fed twice a day with different doses of FC or ChA. Forty larvae were placed in each test chamber for each food condition. Dead larvae were removed daily. Larval death was expressed as a percentage of the total number of individuals subjected to a given condition and compared with the control population receiving a normal diet.

### Statistical analysis

Results are presented as the mean ± SEM of at least three biological replicates. The statistical tests are specified in the figure legends. The data were tested for normal distribution before analysis. In general, the statistical analysis to estimate significance was performed following the recommendation of GraphPad Prism software (version 8.0; GraphPad Software, Inc), as indicated with ∗*P* < 0.05, ∗∗*P* < 0.01, and ∗∗∗*P* < 0.001. Differences were considered significant when *P* < 0.05.

## Results

### ChA is the most prevalent ChE in plasma of CVD patients and in human carotid atheromata

One of the major modifications of LDL trapped in the arterial intima is the oxidation of their PUFA-CEs, which results in the accumulation of so-called “core aldehydes” (25, 27, 41) in the fatty streaks and atheromata. Further oxidation of the core aldehydes results in their conversion to the corresponding ChE (24). Considering the more polar features of ChE, when compared with the core aldehyde precursors, FC and CEs, an increased partitioning of this lipid from the lipid core of the atheroma into its more polar regions and eventually into the plasma is expected. We, therefore, started by determining the plasma levels of ChE in CVD patients, by applying quantitative MS-based shotgun lipidomic analysis.

In order to obtain reliable quantification of ChE, we infused the synthetic standards ChS, ChG (the ChE of glutaric acid), and ChA. We observed that this class of lipids ionize efficiently in the negative ion mode as deprotonated anions. Moreover, we were able to establish the MS/MS fragmentation of the different ChE species and unambiguously detect and quantify them (supplemental Fig. S2A–C). We then assessed whether ChE could be detected and quantified in plasma samples. Increasing amounts of a test plasma sample were extracted together with 100 pmol of ChS, an internal standard that is not normally a component of plasma, and examined in the high-resolution and MS/MS spectra for the ChE mass spectrometric “fingerprint.” Different ChEs, with differing carbon lengths and degrees of unsaturation, were observed. Most importantly, their intensities were correlated with the sample volume (supplemental Fig. S3). The dynamic range and limits of quantification were established by “spiking” increasing amounts of ChS in 2 μl of the plasma sample (supplemental Fig. S4). The dynamic range was 500, and the inferior limit of quantification was 0.5 μM.

**Fig. 1:**
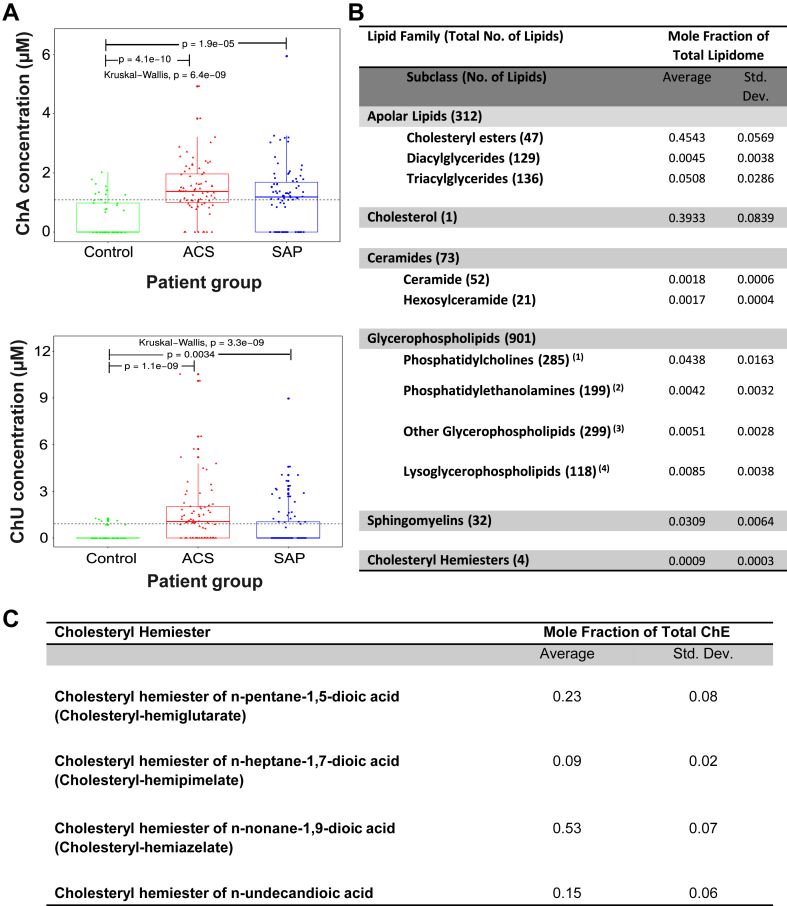
ChA levels are increased in plasma of CVD patients. Boxplots depicting ChA and ChE of 1,11-undecendioic acid (ChU) concentrations across patient groups. A: Kruskal–Wallis one-way analysis of variance was applied to test whether ChA concentration across samples originates from the same distribution. Unpaired two-sample Wilcoxon test was applied as a post hoc test to evaluate significant differences in lipid levels for a patient group versus the control group (indicated by “p”). The horizontal black dashed line indicates the global mean across all samples. Plasma concentration of ChA was obtained by shotgun lipidomics of 74 donors with ACS (including ST-elevation and non-ST-elevation myocardial infarction and unSAP), 71 donors with SAP, and 52 age-matched control cohort. ChA was quantitatively analyzable in 34.6% of the control group, 69% of the SAP group, and 83.8% of the ACS group. B: Lipidomes of the atheromata obtained from six different carotid artery endarterectomy specimens. *1*) Includes ether phosphatidylcholines; *2*) includes ether phosphatidylethanolamines; *3*) includes cardiolipins, phosphatidic acids, phosphatidylglycerols, phosphatidylinositols, and phosphatidyl serines; *4*) includes lysophosphatidic acids, lysophosphatidylcholines, ether-lysophosphatidylcholines; lysophosphatidylethanolamines, ether-lysophosphatidylethanol-amines, lysophosphatidylglycerols; lysophosphatidylinositols, and lysophosphatidylserines. C: ChEs detected in atheromata obtained from the six carotid artery endarterectomy specimens.

In plasma samples, two ChEs were quantitatively analyzable (concentrations >0.5 μM): ChA and a ChE of 1,11-undecanedioic acid (Fig. 1A). ChA was quantifiable in 83.8%, 69%, and 34.6% of acute coronary syndrome (ACS), stable angina pectoris (SAP), and control cohorts, respectively. The ChE of 1,11-undecanedioic acid was only quantifiable in 64%, 31%, and 12% of the ACS, SAP, and control cohorts, respectively. Thus, ChA was the most prevalent of the two ChEs quantitatively detectable in plasma. We also found that average ChA concentrations were significantly higher in ACS and SAP when compared with age-matched controls. The mean differences from control were 1.04 ± 0.15 μM for ACS and 0.73 ± 0.16 μM for SAP. Of note, the highest ChA concentration detected was 5.95 μM (Fig. 1A).

Shotgun lipidomics was also performed on the lipidic content of human atheromata obtained from human carotid endarterectomy specimens. By suspending the carotid endarterectomy cores in water, we observed varying degrees of calcification manifested as granular material that could not be easily homogenized. For this reason, only the “gruel”-like necrotic core was reliably separated from the rest of the samples and used in the analysis. The lipidomic analysis could not, therefore, be related to the total mass of the atheromata. However, our analysis permitted obtaining values of the molar fractions of the total lipid in the “gruel” of the atheromata (Fig. 1B). The obtained results show that CEs and FC together constitute about 85%, triacylglycerides and diacylglycerides constitute about 5%, and polar phospholipids constitute about 10% of the total lipid in the “gruel.” The total ChE content of atheromata is almost 1% of the total polar phospholipid content. In addition, our analysis identified four ChEs, and their relative proportions are shown in Fig. 1C. ChA constitutes a little over 50% of the total ChE content of the atheromata. Interestingly, our findings are well correlated with previous work from other laboratories showing that cholesteryl-9-oxononanoate (the precursor of ChA) was the principal “core aldehyde” in oxLDL (27, 42) and atheromata (25).

Since our previous results (24, 29) have shown that ChEs are able to induce “foam cell” formation and inflammation when macrophages and zebrafish larvae are exposed to ChS, we next investigated the proatherogenic properties of ChA in vitro toward human leucocytes and in vivo toward zebrafish larvae.

### The inflammatory profile of human leucocytes is altered after exposure to ChA

**Fig. 2:**
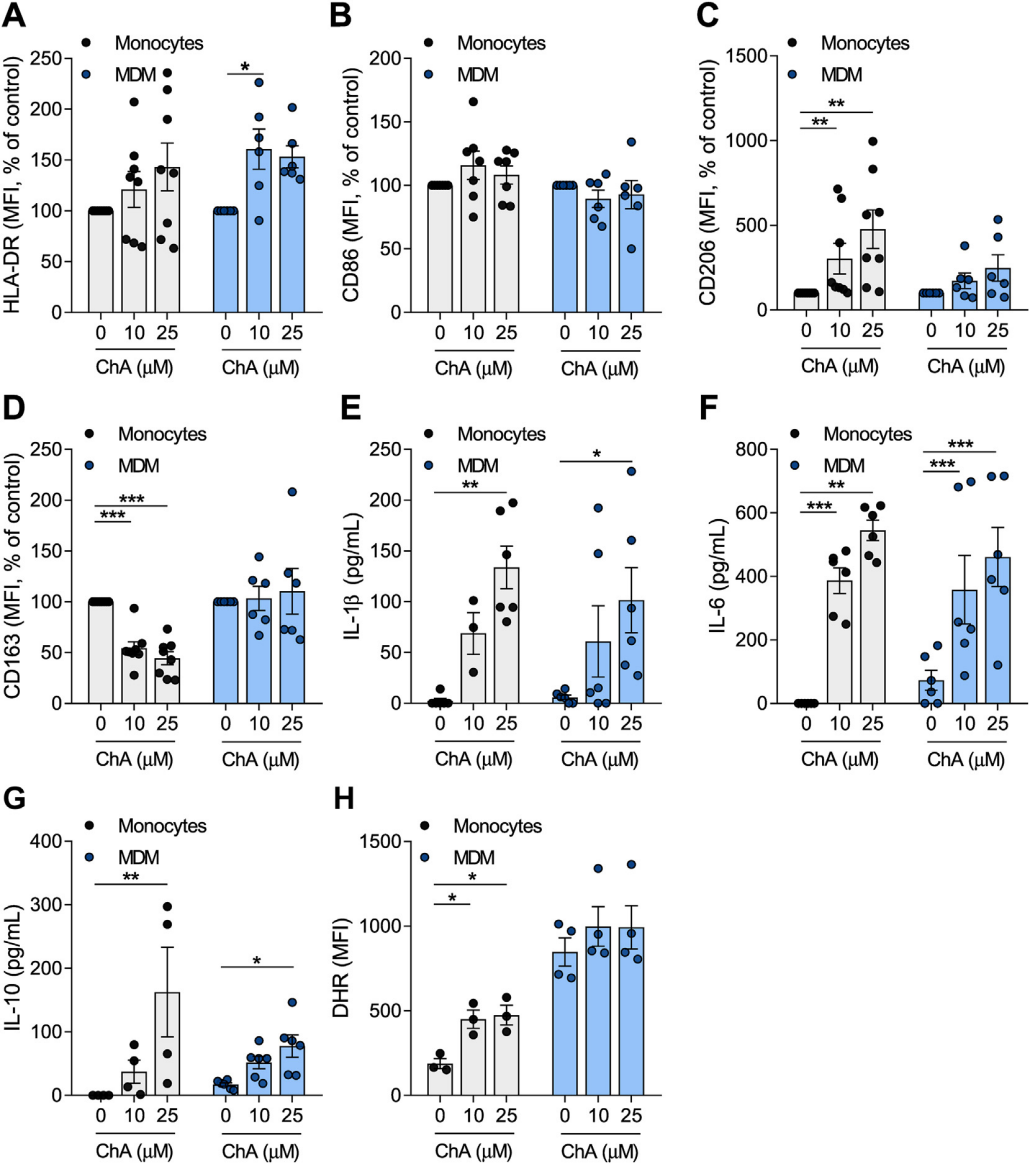
ChA changes the expression of surface markers and the inflammatory profile of human primary monocytes (Mono) and MDMs. Peripheral blood was harvested from human volunteers. Mononuclear leukocytes (peripheral blood mononuclear cells) were isolated from whole blood using histopaque-1077. CD14^+^ cells were purified from total peripheral blood mononuclear cells by immunomagnetic separation. CD14^+^ monocytes were cultured in the presence of ChA (10 μM or 25 μM) or vehicle for 24 h (eight different donors) or for 7 days to obtain MDM (six different donors). Monocytes were differentiated using rM-CSF. After lipid treatment, a representative sample of cells was stained with monoclonal antibodies to determine surface expression of HLA-DR (A), CD86 (B), CD206 (C), and CD163 (D); data represent the mean fluorescence intensity (MFI) ± SEM for all subjects normalized to the control cells. Cell culture supernatants were analyzed by ELISA for IL-1β (E), IL-6 (F), and IL-10 release (G). Values represent the mean ± SEM of cytokine released (in pg/ml). Reactive oxygen species production by monocytes and MDM exposed to ChA (H). ∗*P* < 0.05; ∗∗*P* < 0.01; and ∗∗∗*P* < 0.001 using two-way ANOVA with Tukey’s multiple comparisons test. rM-CSF, recombinant macrophage colony-stimulating factor.

In CVD patients, blood stream leucocytes, such as monocytes and neutrophils, are exposed to several bioactive molecules, among them ChA. In this sense, we studied the effect of ChA on monocyte polarization and on their differentiation into macrophages (human MDMs). Human monocytes (CD14^+^ cells) were incubated for 24 h with vehicle (POPC liposomes, control) and with subtoxic concentrations of ChA (ChA:POPC liposomes, 65:35 M ratio), followed by analysis of specific cell surface markers by flow cytometry. Surface expression levels of HLA-DR and CD86, markers of classically activated monocytes, were not affected by the lipid (Fig. 2A, B). However, alternatively activated surface markers such as CD206 and CD163 were affected by ChA treatment, with ChA-treated monocytes expressing higher levels of CD206 and lower levels of CD163 when compared with control cells (Fig. 2C, D).

Next, we studied the impact of ChA on the differentiation of human monocytes into macrophages. Human monocytes were incubated with M-CSF to induce their differentiation into macrophages in the absence (control) or the presence of ChA at subtoxic concentrations (supplemental Fig. S5A). The percentage of CD14^+^CD16^+^ double-positive cells after differentiation was evaluated (supplemental Fig. S5B). ChA did not affect the monocyte differentiation yield, with the percentage of CD14^int/+^CD16^+^ cells being similar to that found for control cells. In addition, MDM in the presence of ChA revealed an increased expression of HLA-DR and no differences in CD86 when compared with control cells. These proteins are both surface markers of activated (M1) macrophages. In addition, the surface levels of CD163 and CD206, two alternatively activated (M2) markers, revealed no difference in MDM differentiated in the presence of ChA (Fig. 2C, D).

To further characterize the activation stage of monocytes and MDM exposed to ChA, their cytokine profile was evaluated by ELISA. The results shown in Fig. 2E–G demonstrate that ChA-treated monocytes and MDM secreted higher levels of IL-1β, IL-6, and IL-10 than the control cells. Finally, the oxidative burst induced by ChA loaded in immune cells was quantified through the measurement of dihydrorhodamine fluorescence, a reactive oxygen species (ROS) indicator. The results in Fig. 2H demonstrate an increase in ROS production in ChA-treated monocytes, compared with control cells, whereas in MDM, no changes were observed.

Together, these results showed that ChA significantly impacts the monocyte inflammatory phenotype and interferes with the differentiation process of monocytes into macrophages by increasing the expression of inflammatory surface markers, the secretion of inflammatory cytokines, and ROS production.

**Fig. 3:**
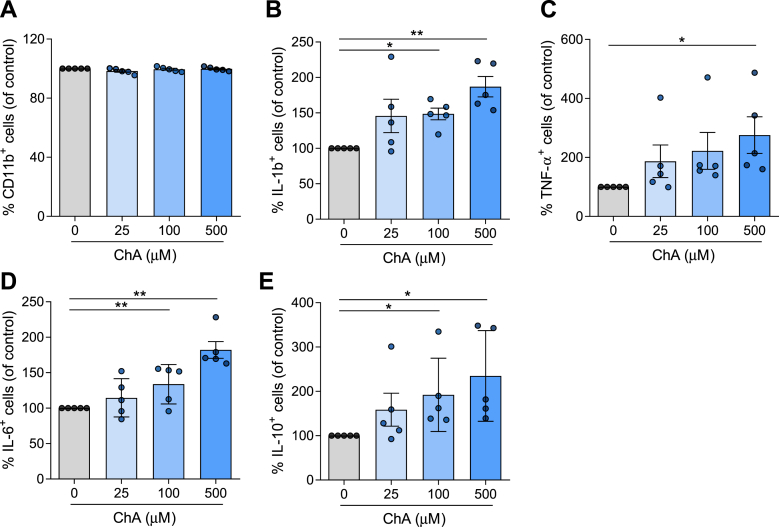
ChA affects the cytokine profile of neutrophils. Neutrophils were harvested from human volunteers (five different donors) using a gradient with histopaque-1177 and 1009. Cells were cultured in the presence of ChA (25, 100, and 500 μM) or vehicle for 4 h. After treatment, neutrophils were stained with monoclonal antibodies to determine the expression of CD11b (A) and cytokines: IL-1β (B), TNF-α (C), IL-6 (D), and IL-10 (E); data represent the percentage of positive cells for each inflammatory marker normalized to the control cells. ∗*P* < 0.05; ∗∗*P* < 0.01 using one-way ANOVA with Dunnett’s multiple comparisons test.

Neutrophils have been implicated in early and advanced atherosclerotic lesions (43). Thus, we assessed the impact of ChA on the inflammatory surface marker, CD11b, and cytokine production in human neutrophils. Human neutrophils were exposed to subtoxic concentrations of ChA (supplemental Fig. S6A) or vehicle (POPC, control) for 4 h. After incubation, cells expressing the surface marker CD11b and the cytokines IL-1β, IL-6, TNF-α, and IL-10 were assessed by flow cytometry. Although, the results presented in Fig. 3A showed that the percentage of cells that were positive for CD11b was not altered by ChA, the percentage of neutrophils positive for IL-1β, IL-6, TNF-α, and IL-10 increased in a ChA concentration-dependent manner (Fig. 3B–E), suggesting that ChA activated neutrophils, leading to a more inflammatory profile. This was reinforced by the observation that IL-1β expression levels, determined when the flow cytometry data were analyzed considering the mean fluorescence intensity, also increase for the highest concentration of ChA (supplemental Fig. S6C). The observation that CD11b surface levels in ChA-treated neutrophils decreased in a dose-dependent manner (supplemental Fig. S6B), when the analysis was performed considering the mean fluorescence intensity, may be because the quantity of this integrin, at the cell surface, is affected by ChA-induced alterations in the fluidity of the membrane lipid bilayer.

Nevertheless, although ChA seems to activate neutrophils only at concentrations 500 times higher than those encountered in the plasma of CVD patients, it is important to have in mind that these in vitro assays have to be performed in short time (4 h), because neutrophils are short-lived cells.

The concentrations of ChA used in this work are far higher than those that any cell might be exposed to in the plasma. Consequently, the results that we report here probably do not always reflect what occurs in the plasma. However, the situation is quite different within the arterial intima for cells that are in direct contact with the atheromal mass. ChA, being an amphiphilic molecule because of the negative charge of the hemiester group, is expected to partition into the polar lipid fraction in a mixture of polar and apolar lipids such as the atheromal lipid mass. This polar lipid fraction, probably saturated with FC, constitutes the surface of atheromal lipid that is exposed to the aqueous phase and which the cells, be they macrophages or smooth muscle cells, will be in contact with. Considering that the molar ratio of ChA to total polar lipid (not including cholesterol) is about 0.5 mol % (Fig. 1B) and given the molar volumes of hydrated glycerophospholipids and cholesterol with which the ChA is associated (44, 45), the corresponding concentration of ChA that the cells in direct contact with the atheromal mass are exposed to is about 3 mM.

In the in vitro experiments, the cells are exposed to a suspension of small (about 100 nm diameter) phospholipid vesicles loaded, to saturation, with ChA. This makes encounter of the cells with the ChA vesicles a second-order random encounter process. The detailed toxicity curve for this process has been very recently published (31) and shows an LD50 value of about 2 mM for the RAW264.7 murine macrophage cell line.

### ChA triggers inflammation in zebrafish larvae

Previous work has shown that zebrafish is a suitable animal model to study high-cholesterol diet-induced accumulation of lipids and inflammation (40, 46). Accordingly, the proteins involved in the transport of dietary fat and inflammatory pathways in zebrafish are conserved relative to mammals (47, 48, 49). Taking this information into account, we decided to pursue our experiments using this animal model to further characterize the inflammatory effects of ChA.

**Fig. 4:**
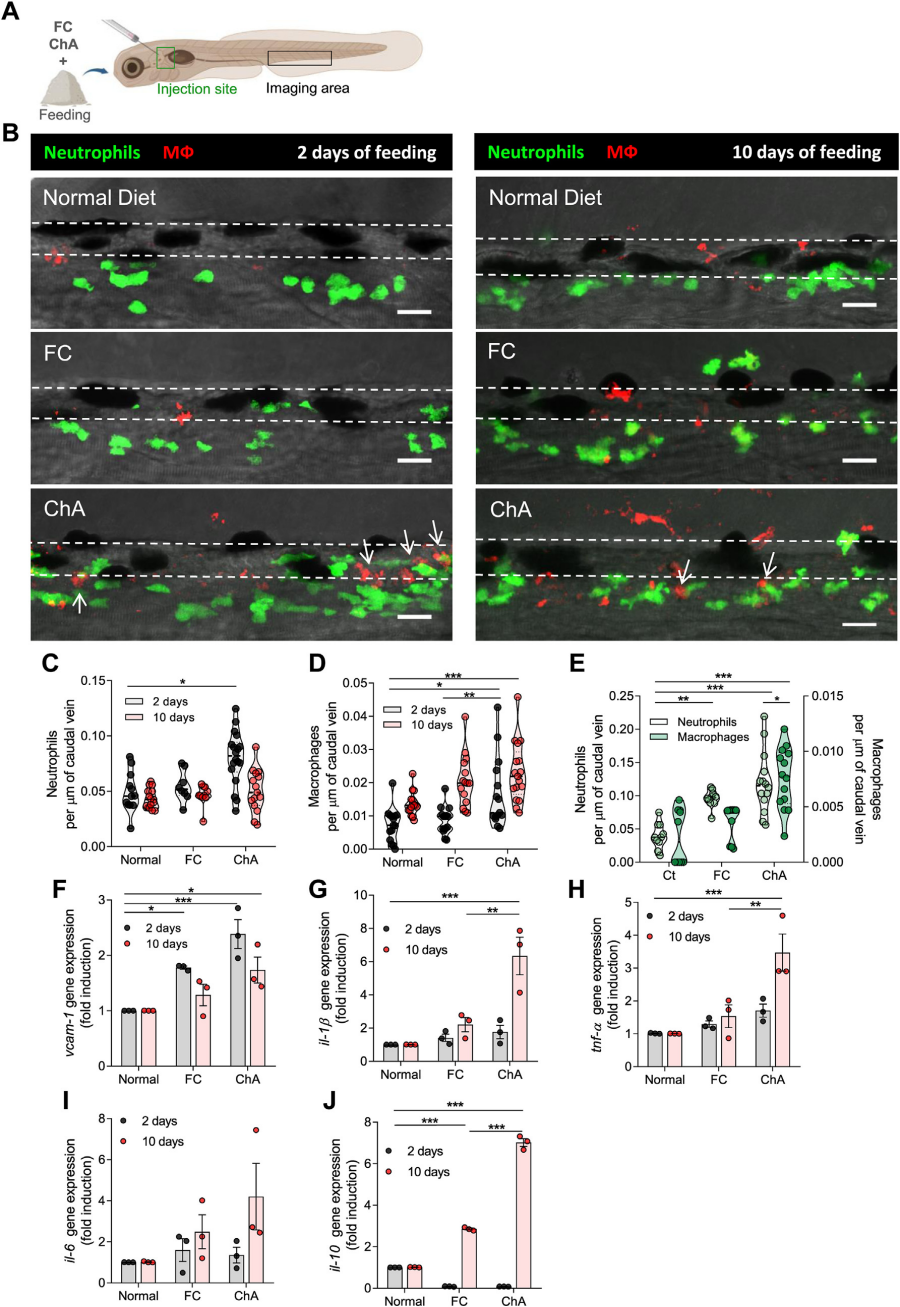
ChA induces infiltration of neutrophils and macrophages in zebrafish caudal vein. A: Schematic representation indicating with rectangles the sites of lipid microinjection and imaging. B: Representative images of the caudal vein of Tg (*mpeg.mCherryCAAX SH378*, *mpx:EGFP i114*) zebrafish larvae fed for 2 or 10 days with normal, FC (4% [w/w] 103 μmol per g of food, positive control), or ChA (3% [w/w], 52 μmol per g of food)-enriched diets and imaged by confocal microscopy. The feeding started at 5 days postfertilization. Images are Z-stacks of fluorescent green (neutrophils) and red (macrophages) cells and the respective bright field. Arrows point to neutrophils and macrophages that are in the close proximity. Dashed lines delineate the caudal vein. Scale bars represent 20 μm. C and D, quantification of neutrophils (green cells, C) and macrophages (red cells, D) after 2 (gray color) or 10 days of feeding (red color). Infiltration of neutrophils occurs earlier than macrophages. The results are shown as mean ± SEM of three independent experiments (in each independent experiment, at least five larvae were analyzed per condition). E: Recruitment of neutrophils (gray color) and macrophages (green color) into the vasculature of Tg (*mpeg.mCherryCAAX SH378, mpx:EGFP i114*) larvae 24 h after the microinjection of POPC (27 μM, vehicle, Ct), FC:POPC (50 μM FC), and ChA:POPC liposomes (50 μM ChA). The graphs represent the mean ± SEM of three independent experiments (at least three larvae in each independent experience were analyzed in each condition). F–J: Quantitative RT-PCR for *vcam-1* (F), *il-1β* (G), *tnf-α* (H), *il-6* (I), and *il-10* (J) after 2 or 10 days of feeding with normal, FC-enriched, or ChA-enriched diets. The values represent the mean ± SEM normalized to the control of three independent experiments (n = 20–30 zebrafish larvae per group); ∗*P* < 0.05 ∗∗*p* ˂ 0.01; ∗∗∗*P* < 0.001 using one-way ANOVA with Dunnett’s multiple comparisons test.

Since one of the hallmarks of inflammation in atherogenesis is the infiltration of innate immune cells into the arterial intima, we assessed whether ChA was sufficient to induce infiltration of myeloid cells into the zebrafish larvae vasculature (Fig. 4A). Zebrafish larvae feeding started at 5 dpf, and as controls, we used fish fed with a normal diet (negative control) and a diet enriched in FC (positive control). *PU.1-EGFP* transgenic zebrafish, whose myeloid cells express GFP, fed for 10 days with FC- or ChA-enriched diets, showed, in a dose-dependent manner, an increase in GFP-labeled cells in the caudal vein in comparison with control animals (visualized in supplemental Fig. S7A and quantified in Figure 7B). Considering that recruitment of myeloid cells with a 3% (w/w) ChA-enriched diet was statistically significant when compared with control, we decided to pursue our experiments using this concentration of lipid. Of note, 3% ChA and 2% FC correspond to the same number of moles of these lipids in the supplemented diet (52 μmol/g of food). As supported by our results (supplemental Fig. S7) and as previously described (29, 40, 46), 4% (w/w) FC (103 μmol per g of food)-supplemented diet was used as positive control. It must be emphasized here that 2% w/w FC, which corresponds, in terms of molarity, to the 3% ChA, did not produce any effect.

To further investigate the kinetics and type of myeloid-cell infiltration, we used the transgenic zebrafish line mpeg.mCherryCAAX SH378, mpx:EGFP i114, that exhibits neutrophils in green (GFP) and macrophages in red (mCherry). As shown in Fig. 4B and quantified in Fig. 4C, D, at 2 days of ChA feeding, neutrophil and macrophage infiltration was significantly increased (*P* < 0.05) in ChA-fed animals when compared with FC-fed and normal diet-fed animals. At 10 days postfeeding, the number of neutrophils was similar in all experimental conditions (Fig. 4C), whereas the number of macrophages increased in ChA-fed animals in comparison with the 2 day feeding (Fig. 4D). These results suggest that the infiltration of neutrophils was transient, whereas the infiltration of macrophages persisted through time.

Since the concentration of lipid in circulation is not controlled during the animal feeding process, we decided to confirm the results obtained previously by directly injecting the lipid into the blood stream of the fish. At 24 h after ChA and FC microinjection, the number of neutrophils in zebrafish caudal vein increased significantly. At this time point, an increase in the number of macrophages was also observed for ChA-microinjected animals but not for the FC-treated animals (Fig. 4E). These results indicate that neutrophil infiltration occurred earlier than macrophage infiltration and that ChA was more inflammatory than FC. Of note, microinjection of embryonic medium with the POPC liposomes alone (the vehicle for FC and ChA, used as a control, Ct) did not impact the number of inflammatory cells in the vasculature, suggesting that the increase in the infiltration of inflammatory cells was indeed only because of FC and ChA (supplemental Fig. S7C–E).

The expression of adhesion molecules can result in the recruitment of inflammatory cells into the vasculature. Therefore, we decided to measure the mRNA levels of the vascular cell adhesion molecule (*vcam-1*) in zebrafish larvae after feeding. The results revealed an increase in *vcam-1* gene expression at 2 and 10 days of ChA feeding (Fig. 4F), which was in accordance with the accumulation of myeloid cells in larvae vascular walls. To gain more insights about the inflammatory effects of ChA using an in vivo system, we next investigated the impact of ChA on the mRNA levels of inflammatory cytokines. Initially, we confirmed the ability of zebrafish larvae to express cytokines at 15 days postfertilization, by incubating zebrafish with lipopolysaccharide, as previously reported (47). An overnight treatment of zebrafish larvae with lipopolysaccharide induced a significant increase in *il-1β*, *tnf-α*, and *il-6* (as well as in *vcam-1*) mRNA expression as compared with untreated control zebrafish (supplemental Fig. S7F–I). Next, we analyzed the levels of these cytokines in larvae fed for 2 and 10 days with normal and FC- and ChA-enriched diets. At 2 days postfeeding, no statistically significant differences were observed in gene expression levels for the inflammatory cytokines tested (Fig. 4G–J). However, at 10 days postfeeding, the results obtained showed an increase on *il-1β* (Fig. 4G), *tnf*-α (Fig. 4H), and *il*-6 (Fig. 4I) expression in ChA-fed larvae. We also observed alterations in *Il-10* (Fig. 4J) mRNA levels induced by FC- and ChA-enriched feeding. These results, obtained using a simple in vivo model, pointed to an inflammatory behavior of ChA.

**Fig. 5:**
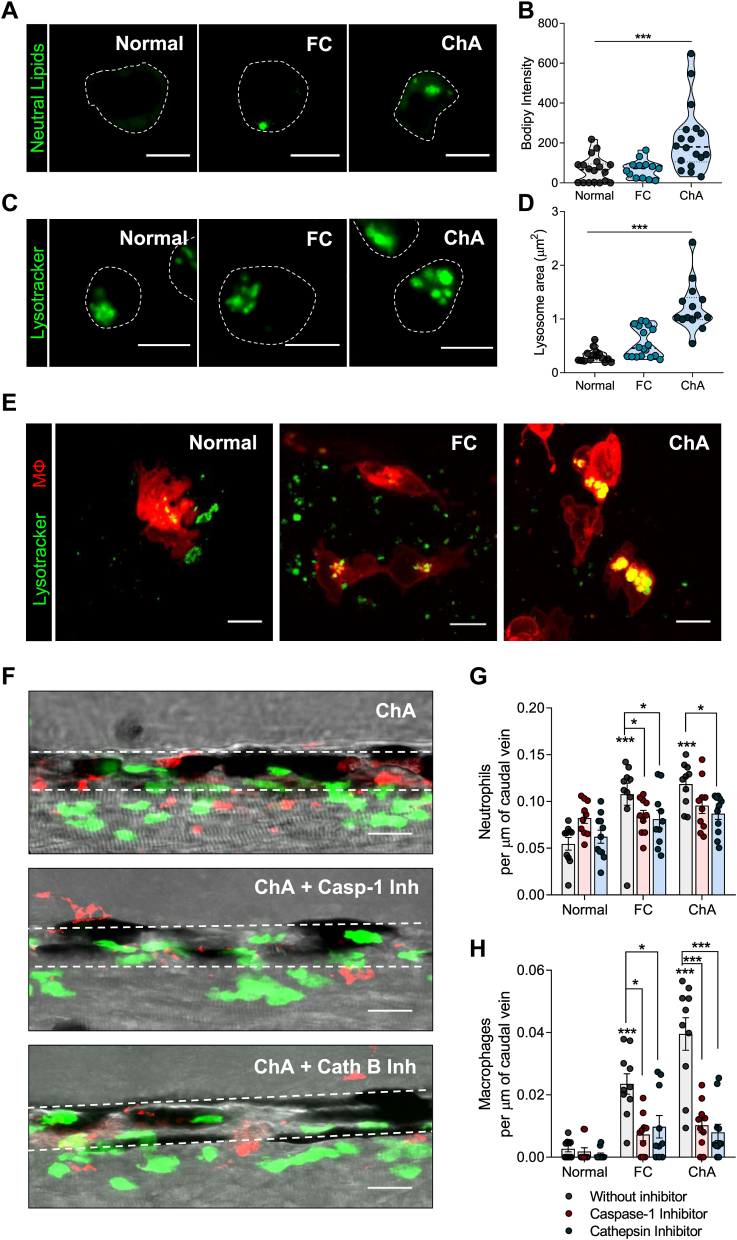
Inflammasome and cathepsin-B inhibition decrease ChA-induced myeloid cell infiltration. A–D: Zebrafish larvae were fed for 10 days with normal, 4% FC-enriched, or 3% ChA-enriched diets, and macrophages were isolated by fluorescence-activated cell sorting (FACS). A: Representative confocal images of macrophages stained with BODIPY 493/503 to visualize neutral lipids. B: Quantification of neutral lipid accumulation on isolated macrophages. C: Representative confocal images of macrophages stained with LysoTracker to visualize lysosomes/acidic organelles. Scale bars represent 5 μm. D: Quantification of the lysosome area on isolated macrophages. In B and D, the results are the mean ± SEM of two independent experiments (at least 10 cells were analyzed per condition); ∗∗∗*P* < 0.001 using one-way ANOVA with Dunnett’s multiple comparisons test. E: In vivo lysosomal imaging of macrophages (m-Cherry) from larvae fed with different diets. Lysosomes were stained with LysoTracker (green). Scale bars represent 5 μm. F–H: Effect of caspase 1 (Casp-1) and cathepsin B (Cath B) inhibitors on neutrophils and macrophage infiltration into the caudal vein of Tg (*mpeg.mCherryCAAX SH378*, *mpx:EGFP i114*) zebrafish larvae fed with normal, 10% FC-enriched, or 14% ChA-enriched food. F*:* Representative confocal images. Images are Z-stacks of fluorescent green (neutrophils) and red (macrophages) cells and the respective bright fields. Dashed lines delineate the caudal vein. Scale bars represent 20 μm. G and H: Quantification of Casp-1 and Cath B inhibitors on neutrophil (G) and macrophage (H) infiltration into the caudal vein. Three independent experiments were performed, and at least three larvae were analyzed per experiment (total of >10 larvae per condition); ∗*P* < 0.05; ∗∗∗*P* < 0.001 using two-way ANOVA with Dunnett’s multiple comparisons test.

We have recently shown that ChA-treated murine macrophages exhibit features of foam cells, namely enlarged dysfunctional lysosomes and exuberant neutral lipid accumulation (31). We, therefore, examined the capacity of ChA to induce neutral lipid accumulation in zebrafish macrophages subjected to ChA feeding for 10 days. The mCherry-positive macrophages were isolated by fluorescence-assisted cell sorting and stained for neutral lipids with BODIPY 493/503. As shown in Fig. 5A and quantified in Fig. 5B, neutral lipid accumulation in macrophages isolated from larvae fed with ChA was much higher than in the larvae fed with FC or with a normal diet (macrophages are outlined by dashed lines). Therefore, we next considered whether these macrophages, from animals that were fed a lipid-enriched diet, would also show evidence of lysosomal dysfunction. Since changes in lysosome area are an indication of their malfunction, we decided to quantify this parameter in macrophages isolated from larvae fed with the different diets by using LysoTracker, a lysosomotropic dye. Remarkably, the results shown in Fig. 5C and quantified in Fig. 5D demonstrated an increase in lysosomal area in macrophages isolated from zebrafish larvae fed with a ChA-enriched diet, compared with the controls. To confirm this latter result, we also evaluated the lysosomal morphology in macrophages in live larvae. As shown in Fig. 5E, macrophages in the vasculature of zebrafish fed with a ChA-enriched diet presented enlarged lysosomes. Interestingly, lysosomal positioning within macrophages from larvae subject to the different types of diet was also impacted. In macrophages of FC-fed animals, lysosomes seemed to be clustered in the perinuclear region, whereas lysosomes in macrophages from ChA-fed animals were located more proximal to the macrophage edges.

Several studies have reported that oxidized lipids from oxLDL, saturated fatty acids, and cholesterol crystals activate the NOD-like receptor protein 3 (NLRP3) inflammasome in macrophages, resulting in IL-1β maturation and secretion (50, 51, 52, 53). Two distinct signals are required for NLRP3 activation: a priming signal, leading to the activation of NF-κB and subsequent upregulation of NLRP3 and pro–interleukin-1β; and an activation signal, which can be provided by a plethora of stimuli that will trigger a series of molecular events culminating in NLRP3 activation. Inflammasome signaling ultimately leads to the activation of the effector caspase-1 that is responsible for the cleavage of pro-IL-1β into IL-1β (supplemental Fig. S8). Signal 2 can be provided by the presence of cathepsin B, a lysosomal protease, in the cytosol, which occurs when lysosomal membrane integrity is lost. Considering the observed increase on *il-1β* expression (Fig. 4G) and the changes in lysosome morphology, we decided to investigate the involvement of the inflammasome as well as the role of lysosomes in our experimental settings. For this purpose, we used caspase-1 and cathepsin B inhibitors. Since lipid-enriched diets led to a significant increase in myeloid cells in the caudal vein of zebrafish, we took advantage of this robust read out to evaluate the role of inflammasome and lysosomes on the inflammation induced by ChA. Zebrafish larvae were pretreated with caspase-1 and cathepsin B inhibitors for 1 h, and then the animals were fed overnight with high doses of FC- and ChA-enriched diets, 10% (w/w) and 14% (w/w), respectively. Our data showed that a single dose of high amounts of FC and ChA was sufficient to induce a significant increase in neutrophil and macrophage recruitment into the vasculature of zebrafish larvae (Fig. 5F–H). Interestingly, treatment either with caspase-1 or cathepsin B inhibitors deeply decreased the recruitment of neutrophils (Fig. 5G) and macrophages (Fig. 5H) induced by FC- and ChA-enriched diets in comparison with control conditions (Fig. 5F–H). We also observed that the effect of cathepsin B inhibitor was more pronounced on the infiltration of macrophages in ChA- than in FC-fed zebrafish, which can be correlated with the observed increase in size and intracellular positioning of lysosomes within macrophages.

These results reveal that, as reported for cholesterol (47), ChA induces the activation of caspase-1 and lysosome membrane permeability loss, which together are involved in the activation of inflammatory pathway resulting in myeloid cell infiltration.

### ChA potentiates lipid accumulation at the sites of the vasculature bifurcation and impacts zebrafish survival

**Fig. 6:**
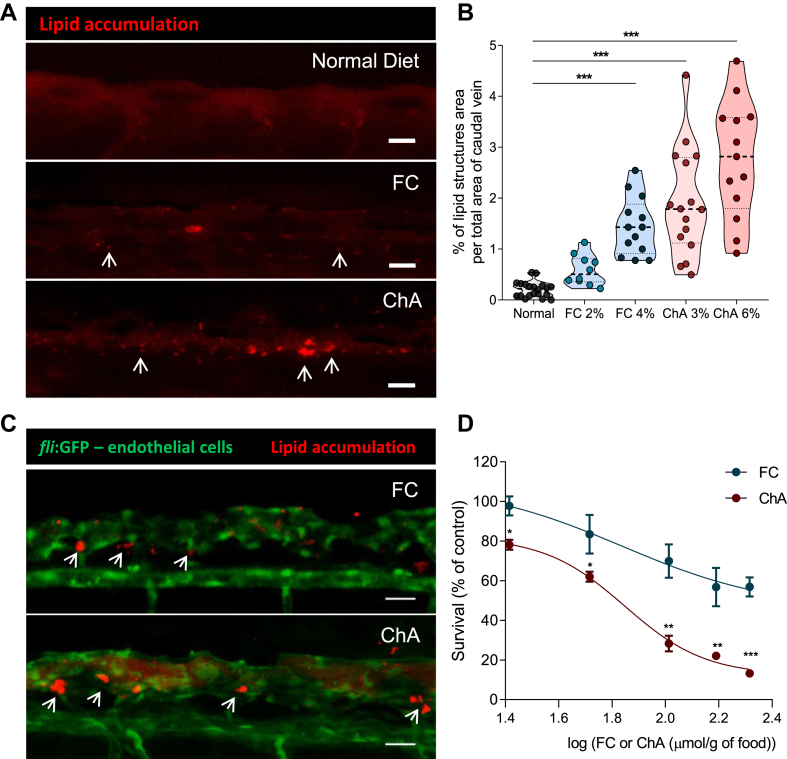
ChA induces lipid accumulation in the vasculature and is toxic to zebrafish larvae. Five days postfertilization, zebrafish larvae were fed for 10 days with normal (in gray), FC-enriched (in blue), or ChA-enriched (in red) diets. A: Confocal z-projection images of fluorescent lipid deposits (in red, indicated by the arrows) of caudal vein of AB larvae. For visualization of lipid structures, diets (normal, 2 or 4% FC-enriched, 3 or 6% ChA-enriched food) were supplemented with 10 μg/g of a red fluorescent CE. Scale bars represent 20 μm. B: Quantification of total lipid structure area in the zebrafish caudal vein. Fluorescent images of at least 10 larvae were quantified per condition. The results are shown as mean ± SEM; ∗∗∗*P* < 0.001 using one-way ANOVA with Dunnett’s multiple comparisons test. C: Z-projection of the caudal vein of *fli1:EGFP* larvae fed as above. Green fluorescence corresponds to endothelial cells (ECs), and red fluorescence corresponds to deposits of lipids localized at the bifurcation sites (arrows). Scale bars represent 20 μm. D: Evaluation of larvae survival as a function of FC (in blue) or ChA (in red) concentration in the diet. Lipid concentration is given by logarithm of 26 μmol/g of food (FC 1%, ChA 1.5%), 52 μmol/g of food (FC 2%, ChA 3%), 103 μmol/g of food (FC 4%, ChA 6%), 155 μmol/g of food (FC 6%, ChA 9%), and 207 μmol/g of food (FC 8%, ChA 12%). Results are mean of two independent experiments (each experiment with 40 larvae). The error bars indicate the SEM and ∗∗*P* < 0.01; ∗∗∗*P* < 0.001 using one-way ANOVA with Tukey’s multiple comparisons test.

Considering the accumulation of neutral lipids in macrophages in ChA-fed zebrafish, we questioned whether neutral lipid accumulation was also observed at the sites of the vasculature bifurcation. We started by analyzing the presence of lipid deposits on the vasculature of AB larvae fed with normal, FC, and ChA diets supplemented with a red fluorescent CE analog (BODIPY 542/563 C11). The caudal veins of the larvae were imaged 10 days after feeding. As previously reported, the observed lipid deposits in caudal veins of zebrafish larvae can be explained by the features of the circulatory system at this stage of larval development (40), when a direct connection between large arteries and veins is observed instead of the capillary network. As shown in Fig. 6A, ChA- and FC-fed larvae showed many focal areas of bright red fluorescence, which we interpreted as lipid accumulation in the vessel wall of the caudal vein. Larvae fed with a normal diet did not exhibit fluorescent lipid structures. The percentage of the area of bright red fluorescent structures in the vessel walls, obtained by confocal Z-stacks of the AB zebrafish larvae, was quantified (Fig. 6B). Our data show that both FC- and ChA-enriched diets increased lipid accumulation in a dose-dependent manner. Remarkably, ChA-induced lipid-accumulation values were much higher than those induced by an equivalent amount of FC, suggesting a more proatherogenic activity. Of note, the lipid accumulation was already significantly increased in larvae fed for 5 days with ChA when compared with the control groups (supplemental Fig. S9).

To further characterize lipid accumulation in the vasculature, *fli1:EGFP* larvae, which constitutively express GFP in the endothelial cells, were fed with different lipid enriched-diets and the lipid tracking probe as described above. We observed that the fluorescence lipid accumulation was subendothelial and localized at sites of blood vessel bifurcation (Fig. 6C). This subendothelial localization is well correlated with what has been described in humans and mice (54, 55).

Finally, we addressed the impact of the ChA-enriched diet on zebrafish larvae survival. For this purpose, zebrafish were exposed to different doses of ChA and FC for 10 days. After this period, viable larvae were counted, and the survival was normalized to the data obtained with animals fed with a normal diet. The results, shown in Fig. 6D, showed that ChA was toxic to zebrafish in a dose-dependent manner. For all doses tested, we also observed higher toxicity of ChA compared with FC. Using the Hill equation, we determined an ED_50_ of 61 and 121 μmol/g, with the minimum percentage of survival (Y_min_) of 12% and 46%, for ChA and FC, respectively.

Together, our results demonstrated that ChA was able to induce lipid accumulation in zebrafish vasculature decreasing the survival of the animals.

## Discussion

Foam cells, lipid-loaded macrophages, and vascular smooth muscle cells, which accumulate in the arterial intima, have long been known to be a characteristic feature of fatty streaks and atheromata. Foam cells may be of two types, a reversible form in which lipid droplets accumulate in the cytoplasm, and an irreversible form that results from lysosomal dysfunction leading to lipid accumulation in dysfunctional lysosomes. Both types are known to occur in atheromata, the reversible form primarily in initial stages (in the so-called “fatty streaks”), and the irreversible form in chronic atheromal lesions. One of the causes of lysosomal dysfunction is cellular uptake of oxLDL particles (56). We have suggested (24) that ChE, the oxidation end products of CEs of PUFA, may be important etiological agents in this process. Several ω-oxoester precursors of ChE have been identified in oxLDL (42) and in the “core aldehyde” fraction of ex vivo samples of human atheromata (25). These oxoesters are expected to be further oxidized in extracellular as well as intracellular environments to their corresponding ChE (24). When formed, ChE may be expected to accumulate at the polar surface of the LDL particles, conferring a negative surface charge to these particles. They would also partition via passive diffusion and transmembrane translocation into all membranes and the cytosol of neighboring cells. Considering that cholesteryl linoleate, the predicted precursor of ChA, is the most abundant CE in plasma (33), we estimated that ChA would be one of the main end products of cholesteryl linoleate oxidation and increased in the plasma of CVD patients. It is, in fact, the most prevalent ChE in plasma of CVD patients and in atheromatous plaque material obtained from human endarterectomy specimens. The plasma levels of ChA are, on the average, about 3-fold higher in CVD patients than in controls. As far as we know, this is the first time that these lipids have been detected and reported to be increased in the plasma lipidome of CVD patients. Here, we show that this newly identified lipid affects the *i*) inflammatory profile of human monocytes, macrophages derived from human monocytes, and neutrophils; *ii*) promotes recruitment of macrophages and neutrophils into the vasculature of zebrafish larvae; *iii*) induces lipid accumulation in the vascular wall, and *iv*) results in a decrease of the animal’s life span.

Monocytes, MDMs and neutrophils treated with ChA exhibit different surface marker profiles when compared with control cells. In our experimental settings, neither monocytes nor macrophages acquire the classically Th-1-polarized M1 or alternatively Th-2-polarized M2 monocyte/macrophage phenotypes (57, 58, 59). These findings suggest distinct monocyte and macrophage phenotypes from the ones already described in the literature. A similar statement can be made for neutrophils. The ChA-driven inflammatory profile in neutrophils is dose dependent, but the concentrations of ChA necessary to observe an inflammatory response were much higher than those required for monocytes or macrophages. This can be explained on the one hand by the fact that neutrophils are short lived, which did not permit long exposure times in our experimental setup. On the other hand, neutrophils could be more resistant to ChA. Indeed, activation of neutrophils, the main type of leucocytes in the blood stream, by low ChA concentrations in the blood stream could be devastating to the CVD patients because of the neutrophil extracellular trap (NET) formation (NETosis) and subsequent thrombus formation (60, 61).

Common to the human leukocytes studied and to the zebrafish larvae is the fact that ChA increases the proinflammatory cytokine IL-1β, a crucial cytokine implicated in the initiation and development of atherosclerosis (62, 63, 64). Thus, ChA could be considered to be a newly identified damage-associated molecular pattern contributing to the inflammatory activity of oxLDL (65, 66). This finding is important since in contrast to oxPL, oxidized cholesterol, and some other oxidized CE products (26, 65, 66, 67, 68, 69, 70, 71), ChE have been almost completely ignored in the literature.

**Fig. 7:**
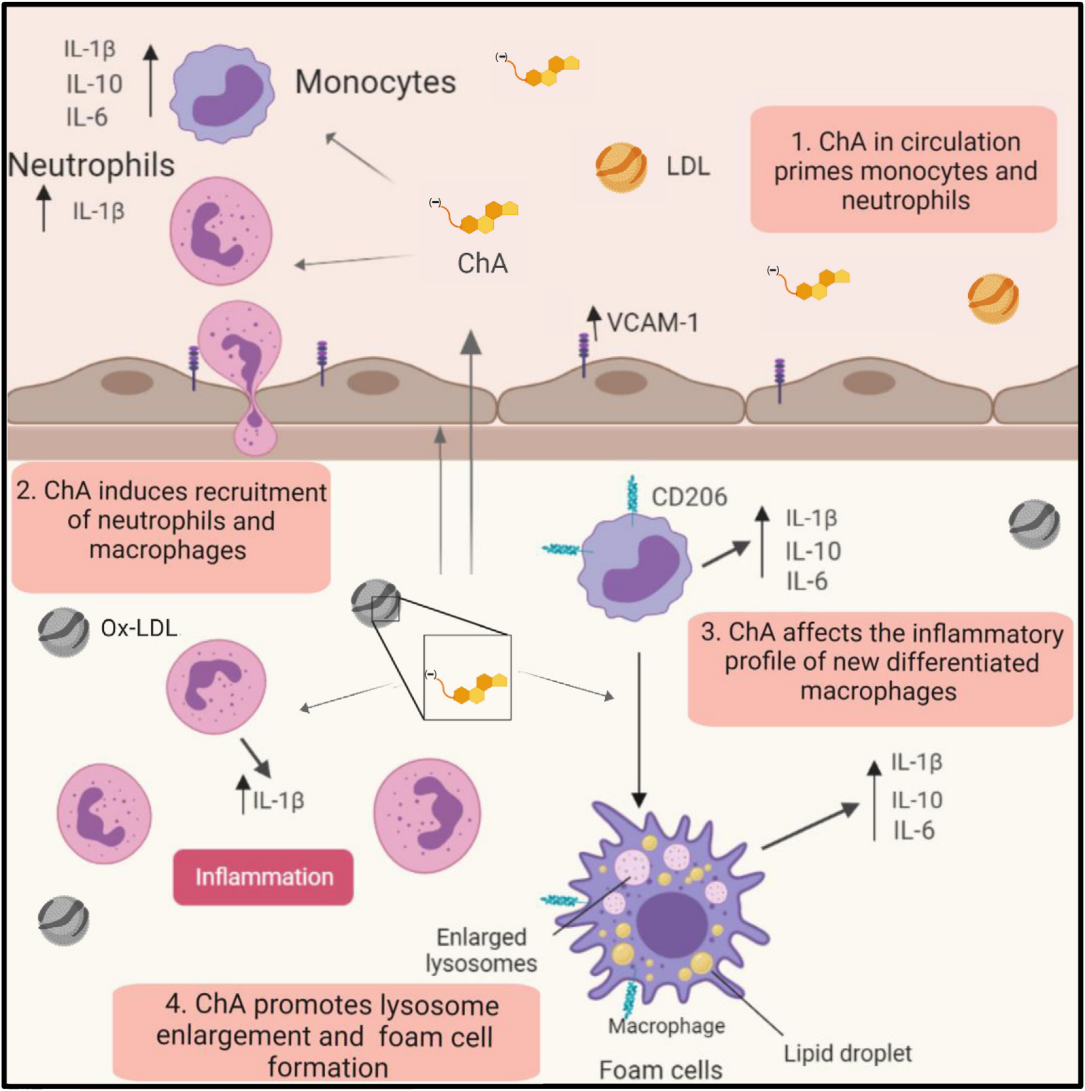
Working model of the proatherogenic properties of ChA. ChA is an end product of cholesteryl linoleate oxidation, generated in the arterial intima. Because of its amphiphilic properties, ChA can be detected in the plasma of CVD patients. The presence of ChA in circulation can imprint an inflammatory phenotype in the circulating monocytes and neutrophils (1), conditioning the immunological response in the arterial intima. ChA promotes the recruitment of innate immune cells, neutrophils, and monocytes into the vasculature (2). Here, neutrophils in the presence of ChA secret IL-1β, which can interfere with monocyte/macrophage priming. Monocytes differentiated in the presence of ChA are activated, increasing the secretion of inflammatory cytokines: IL-1β, IL-6, and IL-10 (3). In activated macrophages, ChA induces lipid accumulation (foam cells) and lysosomal dysfunction, conferring then the second signal necessary for IL-1β secretion mediated by inflammasome activation (4). IL-1β can initiate a propagation loop of the inflammation, increasing the macrophage secretion of IL-6 and TNF-α. On the other hand, dysfunctional lysosomes will decrease the clearance capacity of macrophages, leading to lipid accumulation in the arterial intima.

In vivo ChA triggers infiltration of neutrophils and macrophages into the vasculature of zebrafish larvae (Fig. 7). This is a key event in the pathogenesis of atherosclerosis since the recruitment of these cells may result in exacerbated oxidation of LDL, setting the stage for catalytic expansion of the atherosclerotic lesion and the full-blown spectrum of atherosclerosis (72). In our experimental settings, neutrophil infiltration is transient. On the contrary, macrophages accrue with time within the vasculature. This feature is compatible with a nonresolved inflammation state. Macrophage accumulation coincides with an increase on *il-1β* transcripts, and pharmacological inhibition of the inflammasome dampens the infiltration of inflammatory cells. This can be explained by the fact that rise in inflammatory mediators leads to additional rounds of recruitment of monocytes and neutrophils (73). In addition, similarly to what has been described in the literature (47, 50, 53), loss of lysosome membrane integrity seems also to be the culprit of inflammation. Most interestingly, inhibition of the lysosomal cathepsin B activity leads to a reduction of neutrophils and macrophages in the vasculature, suggesting the involvement of lysosomes in ChA-mediated inflammation by IL-1β production. Thus, we can envision that lipid accumulation in dysfunctional lysosomes causes loss of lysosome membrane integrity, with cathepsin B leakage into the cytosol and subsequent inflammasome activation and IL-1β secretion (Fig. 7).

Vascular inflammation is accompanied by vascular lipid accumulation. Using transgenic zebrafish with GFP-labeled endothelial cells fed with ChA, we observed an increase in lipid accumulation at the caudal vein bifurcations, where turbulent blood flow is expected to occur. Our results are in accordance with those observed for zebrafish fed with FC- and ChS-enriched diets (29, 40), as well as with published data in humans and mice, where a higher propensity of lipid-driven disease is observed in arterial branches with disturbed flow (54, 55). Finally, inflammation and lipid accumulation impact zebrafish larvae life expectancy, ChA being more lethal than FC (40).

Thus, in this comprehensive study, we showed in vivo and in vitro inflammatory and atherogenic properties of ChA, a newly identified and quantified oxidized lipid in CVD patients. In the future, a better understanding of the molecular mechanisms underlying the biological effects of ChA may identify specific targets for future therapies, offering better focused and potentially personalized treatment of CVD. Moreover, a further analysis on the diagnostic and/or prognostic potential of ChA concentration in plasma of patients may provide novel clinical tools to tackle CVD.

## Data availability

All data are contained within the article.

## Supplemental data

This article contains supplemental data.

## Conflict of interest

The authors declare that they have no conflicts of interest with the contents of this article.
