# Supplementary material for: Cholesteryl hemiazelate identified in CVD patients causes in vitro and in vivo inflammation
Source: J Lipid Res. 2023 Jul 21;64(9):100419. doi: 10.1016/j.jlr.2023.100419 (PMC10450993; doi:10.1016/j.jlr.2023.100419)
Supplement: Supplemental Table 1 [file mmc3.pdf]

Supplementary Table 1: Baseline characteristics of the analyzed lipid cohort

	Control (N=52)	SAP (N=71)	ACS (N=74)	CEA (N=6)
**Sex**				
**Female**	34 (65.4%)	21 (29.6%)	18 (24,3%)	1 (16.7%)
**Male**	18 (34.6%)	50 (70.4%)	56 (75.7%)	5 (83.3%)
**Age**				
**Mean (SD)**	55.9 (13.5)	67.1 (10.9)	66.3 (12.7)	75.8 (2.3)
**Range**	36 - 81	33-86	36-92	73 -79
**Weight**				
**Mean (SD)**	68 (14)	77 (13)	77 (14)	74 (4)
**Height**				
**Mean (SD)**	167 (8.9)	166 (8.5)	170 (7.8)	166 (4)
**BMI**				
**Mean (SD)**	25 (3.9)	28 (4)	28 (3.9)	27(2)
**Statin Use**				
**No**	46 (88.5%)	25 (35%)	40 (54%)	4 (67%)
**Yes**	6 (11.5%)	46 (65%)	34 (46,0%)	2 (33%)
**Plasma lipid levels**				
**CE (µM)**	4805 ± 963	3973 ± 1010	3821 ± 1015	5465 ± 1549
**FC (µM)**	1280 ± 255	1257 ± 404	1237 ± 377	1354 ± 285

SAP, stable angina pectoris; ACS, acute coronary sindrome; CEA, human carotid endarterectomy specimens

CE, cholesteryl esters; FC, free cholesterol
